# WNK4 limits distal calcium losses following acute furosemide treatment

**DOI:** 10.14814/phy2.14195

**Published:** 2019-09-08

**Authors:** Mohammed Z. Ferdaus, Brittany D. K. Gratreak, Lauren Miller, Jinge Si, James A. McCormick, Chao‐Ling Yang, David H. Ellison, Andrew S. Terker

**Affiliations:** ^1^ Division of Nephrology Oregon Health and Science University Portland Oregon; ^2^ Oregon Clinical and Translational Research Institute, Oregon Health and Science University Portland Oregon; ^3^ Division of Nephrology Vanderbilt University Medical Center Nashville Tennessee

**Keywords:** Calcium, distal convoluted tubule, thick ascending limb, WNK4

## Abstract

The distal nephron is essential for calcium homeostasis. This is evidenced by disordered calcium transport following disrupted distal nephron function occurring in salt‐wasting tubulopathies or with diuretic use. A plethora of studies support a role for WNK4 in thick ascending limb (TAL) and distal convoluted tubule ion transport with most studies focusing on sodium transport. Little is known about the *in vivo* role of WNK4 in regulating calcium homeostsis. Here, we investigated the role of WNK4 in regulating distal nephron calcium transport using WNK4 knockout animals (WNK4^−/−^). As has been shown previously, we found that baseline urinary calcium levels are normal following WNK4 deletion. Following acute treatment with the loop diuretic, furosemide, which causes hypercalciuria through TAL inhibition, WNK4^−/−^ animals demonstrated increased calcium wasting compared with wild‐type controls. WNK4^−/−^ animals had decreased TRPV5 expression along DCT2 supporting a mechanistic role for this calcium channel in the increased calciuresis. As this supported the hypothesis that WNK4^−/−^ animals have a tendency toward calcium wasting under stress, we tested the effects of a calcium‐deplete diet on urinary calcium excretion. Urinary calcium excretion and plasma ionized calcium levels were not different between control and knockout animals following consumption of a calcium‐deplete diet. Our data show that WNK4, via regulation of TRPV5, limits distal calcium losses following acute treatment with furosemide; however, WNK4 deletion does not affect the chronic renal response to dietary calcium depletion. Our data reveal an *in vivo* role for WNK4 in distal nephron calcium handling that is important for fine‐tuning calcium reabsorption.

## Introduction

The distal nephron is essential for calcium homeostasis. Genetic disruption of distal nephron electroneutral cation‐coupled chloride cotransporters, seen in Mendelian salt‐wasting tubulopathies, impairs renal calcium handling. Thick ascending limb (TAL) dysfunction, seen in Bartter syndrome, and distal convoluted tubule (DCT) dysfunction, seen in Gitelman syndrome, cause hypercalciuria and hypocalciuria, respectively. Pharmacologic calciuretic phenocopies are observed with loop and thiazide diuretics, which specifically target these nephron segments, further emphasizing the importance of the TAL and DCT in calcium homeostasis.

Along the TAL, calcium is reabsorbed primarily via a paracellular pathway (Ferre et al. 2012) driven by the lumen positive potential maintained by apical activity of the Na^+^‐K^+^‐2Cl^‐^ cotransporter (NKCC2) and the renal outer medullary K^+^ channel (ROMK). Along the DCT, the reabsorptive process is transcellular with calcium entering through the apical calcium channel, transient receptor potential cation channel subfamily V member 5 (TRPV5), and exiting via the basolateral plasma membrane calcium ATPase (PMCA) and Na^+^/Ca^2+^ exchanger (NCX) (Boros et al. 2009; Dimke et al. 2011).

The electroneutral cation‐coupled chloride cotransporters NKCC2 and Na^+^‐Cl^‐^ cotransporter (NCC) are the predominant routes for transcellular sodium reabsorption along the TAL and DCT respectively. For both, cotransporter activity is regulated by a molecular network comprising With no lysine (WNK) kinases and their downstream targets Ste20p‐related proline alanine‐rich kinase (SPAK), and oxidative stress responsive kinase 1 (OxSR1). Studies using WNK4 null animals have demonstrated the importance of the WNK4‐SPAK/OxSR1‐NCC pathway along the DCT for regulation of sodium and potassium homeostasis in vivo (Castaneda‐Bueno et al. (2012); Takahashi et al. 2014). Using this same model, we recently reported that WNK4 is also important for sodium transport along the TAL (Terker et al. 2018).

Multiple studies have also implicated WNK4 in the molecular regulation of renal calcium homeostasis. Most notably, mutations in WNK4 cause the Mendelian disease Familial hyperkalemia and hypertension (FHHt), which results in a hyperactive NCC and clinically presents with hypercalciuria and low bone mineral density in addition to hypertension, hyperkalemia, and metabolic acidosis (Mayan et al. 2002). These findings were recapitulated in a mouse model of FHHt (Yang et al. 2010). In vitro studies have shown WNK4 regulates TRPV5 (Jiang et al. 2007; Jiang et al. 2008; Hoover et al. 2016) suggesting a mechanistic explanation for these *in vivo* observations. Moreover, TRPV5 knockout animals exhibit a renal calcium‐wasting phenotype, and human sequence variants in this gene associate with recurrent kidney stones (Oddsson et al. 2015), supporting its importance in renal calcium handling.

Here, we investigate the role of WNK4 in calcium transport along the TAL and DCT using WNK4^−/−^ animals. While baseline urinary calcium levels are known to be normal in WNK4^−/−^ animals (Castaneda‐Bueno et al. 2012; Terker et al. 2018), we have reported differences in urine calcium excretion between controls and knockout animals in response to dietary stress (Terker et al. 2018). We sought to determine the role of WNK4 in calcium handling under conditions known to perturb calcium homeostasis. We show that WNK4 is essential to limit calcium losses following acute administration of the loop diuretic furosemide, likely through regulation of TRPV5. Despite this clear role for WNK4 in preventing acute excessive calcium loss, we subsequently demonstrate that the renal response to chronic dietary calcium depletion is preserved in the absence of WNK4.

## Methods

### Animals

*wnk4* ^−/−^ mice were rederived from cryopreserved sperm (Castaneda‐Bueno et al. 2012) at Charles River onto a C57Bl/6NCrl background. Animal studies were approved by Oregon Health and Science University Institutional Animal Care and Use Committee. Wild‐type littermates were used as control animals.

### Dietary manipulation

For baseline urine collection, animals were fed normal diet (TestDiet AIN‐93G 0.36% K^+^, 0.51% Ca^2+^ and adjusted to 0.49% Na^+^). For calcium‐deficient diet study, animals were fed Teklad low calcium diet (TD.95027, Envigo, 0.01% Ca^2+^) supplemented with CaCl_2_ to 0.51% Ca^2+^ for baseline urine collection followed by the Ca^2+^‐deficient diet for the subsequent 4 days. All animals used for dietary experiments were female.

### Urine collection for dietary study

Animals were acclimated to metabolic cages (Hatteras Instruments MMC100) for 2 days before urine collection. Animals were fed a gelled diet (calcium‐deficient diet with or without supplemented CaCl_2_ as described above depending on experimental conditions) and had free access to water. Urine was collected under water‐saturated light mineral oil after 24 h. Urine was collected following consumption of baseline diet and three days of consumption of calcium‐deplete diet. Urine Ca^2+^ was assayed using the *o*‐cresolphthalein method (Pointe Scientific C7503).

### Blood analysis

Animals were sacrificed following the fourth day of consumption of calcium‐deplete diet (Day 5 of experiment). Whole blood was collected via cardiac puncture. Plasma electrolytes and hematocrit values were obtained by iSTAT just after collection by loading whole blood into a chem 8 cartridge (Abbot Point of Care).

### Furosemide response test

Animals were injected intraperitoneally with vehicle (1.2% ethanolamine in normal saline), then placed in metabolic cages and urine was collected for 3 h. On a different day, the same animals were injected with furosemide (25 mg/kg body weight) in vehicle, followed by 3 h urine collection. Hydrochlorothiazide (HCTZ) was injected daily at 25 mg/kg for 5 consecutive days. On day 5, the furosemide response test was performed as above with either vehicle or furosemide (25 mg/kg) injected 1 h following the HCTZ injection. Animal sexes included: 2 male WNK4^+/+^, 4 female WNK4^+/+^, 3 male WNK4^−/−^, and 4 female WNK4^−/−^ for experiments with furosemide alone; 4 male WNK4^+/+^, 3 female WNK4^+/+^, 1 male WNK4^−/−^, and 6 female WNK4^−/−^ for experiments with furosemide and HCTZ.

### Immunofluorescence

Animals were injected with anesthesia cocktail (ketamine/xylazine/acepromazine, 50/5/0.5 mg/kg), and under deep anesthesia animals were perfusion fixed with 4% paraformaldehyde. After cryoprotection in 800 mosmol/L sucrose and freezing in Optimal Temperature Cutting (OCT) compound, 5‐μm sections were cut. Sections were incubated overnight at 4°C with anti‐anti‐TRPV5 (1:100, Alomone) or anti‐calbindin (1:500, Swant). Sections were incubated in secondary antibody at 1:2000 for 1 h at room temperature [Alexa Fluor 488 donkey anti‐rabbit (Life Technologies A21206) or Alexa Fluor 555 donkey anti‐mouse (Life Technologies A31570)]. All sections were mounted with ProLong Diamond Antifade Mountant (ThermoFisher Scientific P36970). Images were captured with a ZEISS AXIO Imager M2 fluorescent microscope.

### Statistical analyses

The null hypothesis was tested using unpaired Mann–Whitney *U*‐test or two‐way analysis of variance (ANOVA) (with or without repeated measures as indicated in figure legends) using GraphPad Prism 8 as indicated in the figure legends.

## Results

WNK4^−/−^ mice have normal urine calcium excretion at baseline (Castaneda‐Bueno et al. 2012; Terker et al. 2018), but the role of WNK4 in regulating calcium handling under conditions known to perturb calcium homeostasis remains unclear. The loop diuretic furosemide causes hypercalciuria, both chronically and acutely. We reported decreased furosemide‐induced natriuresis in WNK4^−/−^ mice (Terker et al. 2018). To determine effects of WNK4 deletion on furosemide‐induced calcium excretion, we quantified urine calcium following acute furosemide treatment.

**Figure 1:**
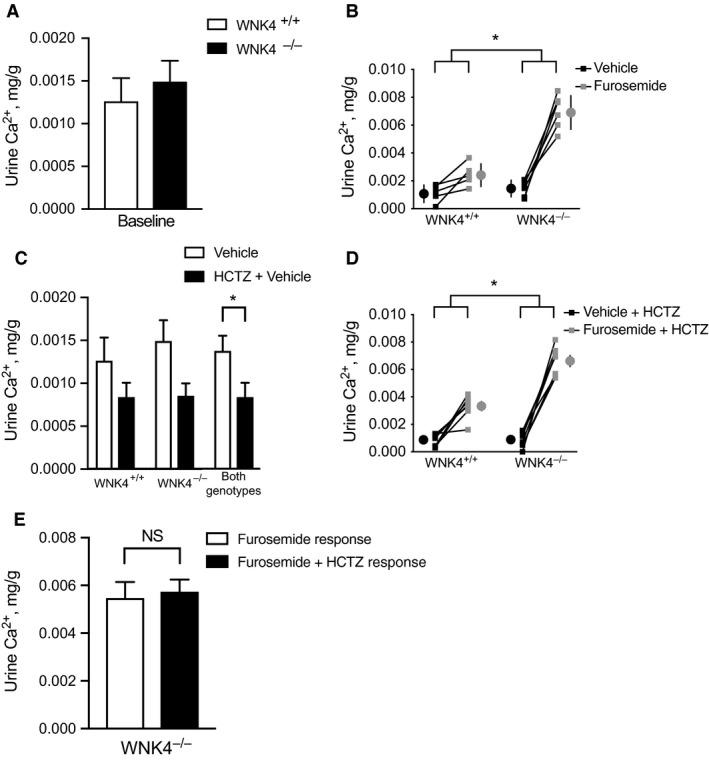
Effects of acute furosemide administration on 3‐h urine calcium excretion in control (WNK4^+/+^) and WNK4 knockout (WNK4^−/−^) animals. (A) Baseline urine calcium excretion was not different between control and WNK4^−/−^ animals following vehicle administration. (B) Acute furosemide administration increased urine calcium levels in both genotypes. The effect was greater in WNK4^−/−^ animals. Large circles represent means ± SEM. (C) Hydrochlorothiazide (HCTZ) administration decreased 3‐h urine calcium excretion. Data trended toward statistical significance when analyzed for each genotype separately. When genotypes were combined, the difference between vehicle and HCTZ achieved statistical significance. (D) Acute furosemide administration increased urine calcium levels in both genotypes in the presence of HCTZ. Again, effects were greater in WNK4^−/−^ animals. Large circles represent means ± SEM. (E) HCTZ did not affect the calciuretic response to acute furosemide treatment in WNK4^−/−^ animals. *N* = 5–7 per group. Sexes used were: 2 male WNK4^+/+^, 4 female WNK4^+/+^, 3 male WNK4^−/−^, and 4 female WNK4^+−/−^ for 1a, b; 4 male WNK4^+/+^, 3 female WNK4^+/+^, 1 male WNK4^−/−^, and 6 female WNK4^+−/−^ for 1c, d. Data are displayed as mean ± SEM. For A, C, and E, effects compared with Mann–Whitney *U*‐test. For B and D, effects compared with two‐way ANOVA. *P < 0.05. For B and D interaction between genotype and treatment was statistically significant.

Consistent with previous reports, baseline urine calcium excretion was not different between control and WNK4^−/−^ mice in this model (Fig. 1A). As expected, furosemide caused a calciuresis in control animals. This effect was 3.5 times greater in WNK4^−/−^ animals (Fig. 1A). To determine if this effect was dependent on NCC function, we treated animals with hydrochlorothiazide (HCTZ) for five days prior to acute furosemide treatment. HCTZ induced a trend toward reduced baseline urine calcium excretion in both genotypes; this effect was statistically significant when data for both genotypes were pooled (Fig. 1C). The increased calciuresis seen in WNK4^−/−^ animals following furosemide administration was not affected by HCTZ treatment (Fig. D and E).

**Figure 2:**
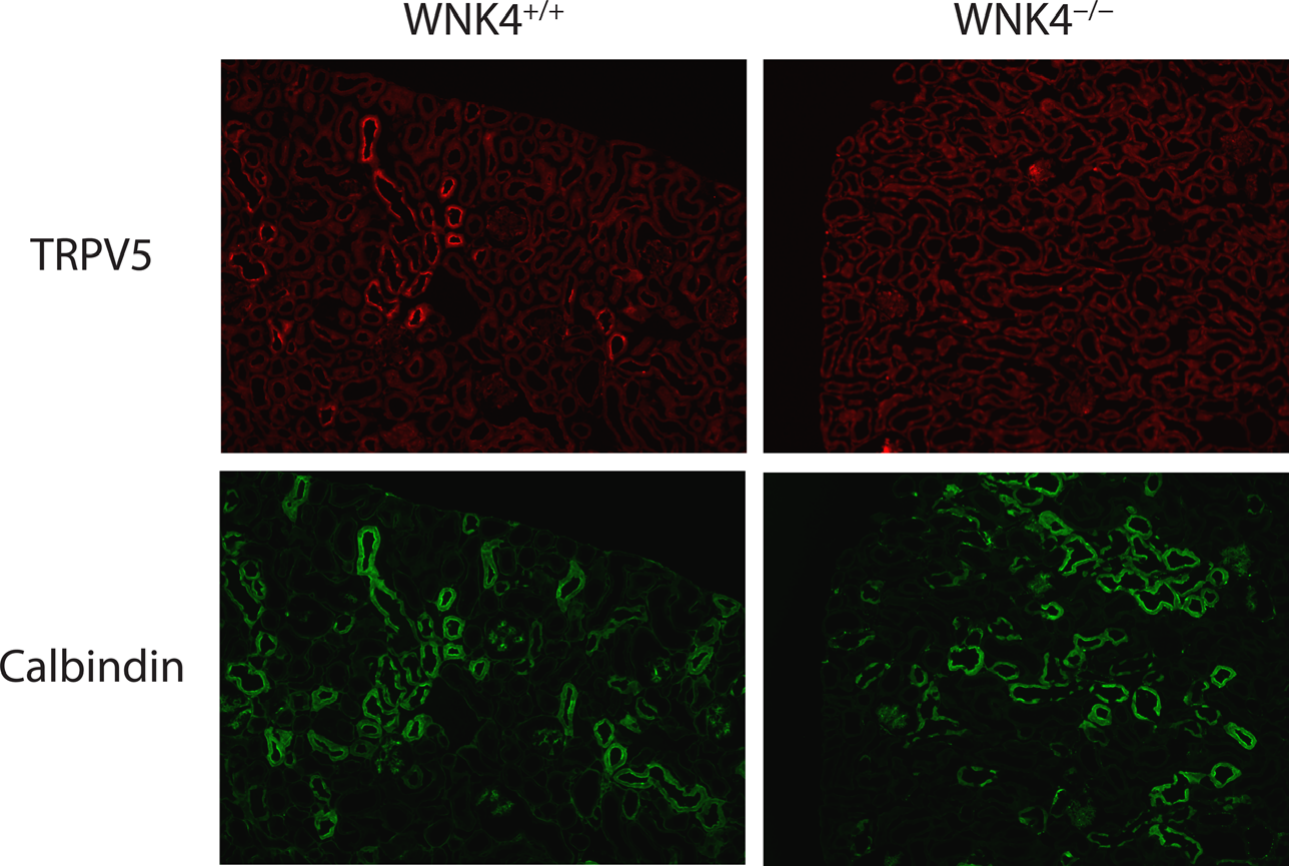
Effects of WNK4 deletion on TRPV5 expression in DCT2. TRPV5 expression along the DCT2 is substantially reduced in WNK4 knockout animals (WNK4^−/−^) compared to controls (WNK4^+/+^). DCT2 is identified by calbindin immunopositive tubules. Costained sections are presented. Images were obtained for *n* = 4 for each genotype and representative images are shown.

Previous reports have shown that WNK4 increases the activity of TRPV5(Jiang et al. 2007; Jiang et al. 2008; Hoover et al. 2016). We tested the hypothesis that the observed increased calciuresis occurs because WNK4^−/−^ animals have reduced the TRPV5 activity. This may result in a failure to reabsorb excess calcium distally following acute furosemide treatment. Consistent with this, WNK4^−/−^ animals had substantially decreased baseline TRPV5 expression along the DCT2 (Fig. 2) as shown by colocalization with calbindin, a known DCT2 marker (Nijenhuis et al. 2003).

**Figure 3:**
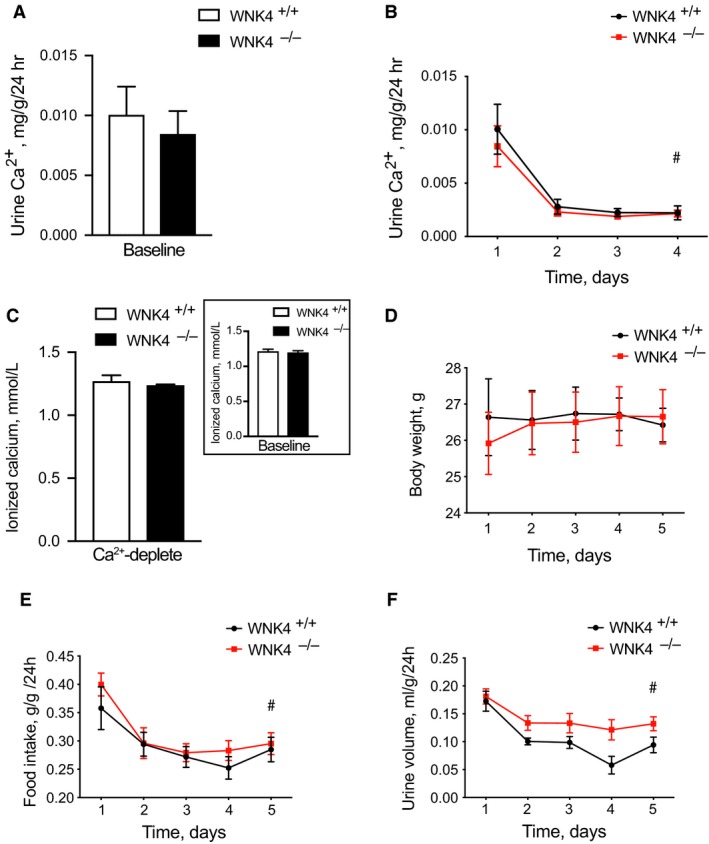
Effects of dietary calcium depletion on urine calcium excretion in control (WNK4^+/+^) and WNK4 knockout (WNK4^−/−^) animals. (A) Baseline urine calcium excretion was not different between control and WNK4^−/−^ animals following consumption of calcium‐replete diet. (B) Urine calcium excretion decreased in control and WNK4^−/−^ animals in response to dietary calcium depletion. The effect was not different between genotypes. (C) Plasma ionized calcium levels did not differ by genotype following consumption of a calcium‐deplete diet for 4 days. Inset shows that values were comparable to baseline ionized calcium levels measured in a separate group of animals maintained on a calcium replete diet. (D) Body weight did not change in either genotype following consumption of a calcium‐deplete diet for 4 days. (E) Daily food consumption decreased in control and WNK4^−/−^ animals following consumption of a calcium‐deplete diet. The effect was not different between genotypes. (F) Daily urine volume decreased in control and WNK4^−/−^ animals following consumption of a calcium‐deplete diet. The effect was not different between genotypes. *N* = 4 for WNK4^+/+^, *N* = 5 for WNK4^−/−^. All animals were female. Data are displayed as mean ± SEM. For A and C, effects compared with Mann–Whitney *U*‐test. For B and D–F, effects compared with two‐way ANOVA with repeated measures. #*P* < 0.05 for effect over time in both genotypes. Interaction was not statistically significant.

Having demonstrated WNK4^−/−^ animals have dysregulated calcium transport following pharmacologic stress, we next tested the hypothesis that WNK4^−/−^ animals waste calcium under dietary stress. To do this, we determined effects of dietary calcium depletion on control and WNK4^−/−^ animals. Data were collected on a normal calcium diet for 24 h followed by three days (experimental days 2–4) of dietary calcium depletion. Again, 24‐h urine calcium excretion did not differ between genotypes at baseline on a normal calcium diet (Fig. 3A). Under calcium‐deplete conditions, both groups reduced 24‐h urine calcium excretion by over 70% (Fig. 3A). There was no difference in response between genotypes. Moreover, blood ionized calcium levels were not different between genotypes at the end of the experiment (Fig. 3A). Note, these ionized calcium levels did not differ from baseline levels when compared with a different group of animals consuming a normal calcium diet (Fig. 3A inset). Body weight did not differ significantly between groups and did not change over time (Fig. 3A). Food consumption decreased in both groups to an equivalent degree (Fig. 3E). Urine volume also decreased in both groups, and while there appeared to be a trend toward a greater decrease in control animals, the interaction was not statistically significant (Fig. 3F).

Electrolytes following calcium depletion were consistent with the previously reported baseline WNK4 knockout phenotype (Castaneda‐Bueno et al. 2012; Terker et al. 2018), as WNK4^−/−^ animals demonstrated hypochloremic metabolic alkalosis, although differences in bicarbonate were not statistically significant (Table 1).

**Table 1:** Plasma electrolytes and hematocrit in control (WNK4^+/+^) and WNK4 knockout (WNK4^−/−^) animals following dietary calcium depletion.

	WNK4^+/+^	WNK4^−/−^	*P*‐value
Na (mmol/L)	145.3	145	0.74
K (mmol/L)	3.67	3.22	0.14
Cl (mmol/L)	112	105	0.005
HCO_3_^‐^ (mmol/L)	23.7	27	0.1
iCa (mmol/L)	1.27	1.24	0.35
Hct (%)	39.7	42.3	0.18

## Discussion

Multiple studies have confirmed that WNK4^−/−^ animals have normal urine calcium excretion at baseline (Castaneda‐Bueno et al. 2012; Terker et al. 2018). Urinary calcium differentiates Gitelman from Bartter syndrome, with hypocalciuria seen in Gitelman patients and normo‐ or hypercalciuria seen in Bartter patients. Baseline normocalciuria following WNK4 deletion is a feature that resembles type 3 Bartter syndrome. This variant of Bartter syndrome is caused by mutations in the chloride voltage‐gated channel Kb (CLCNKB) (Simon et al. 1997), which results in dysfunction along both the DCT and TAL (Grill et al. 2016). Normocalciuria in this syndrome is thought to arise from a “balancing” of the hypercalciuria and hypocalciuria generated from sequential nephron dysfunction.

Both DCT and TAL dysfunction are also observed following WNK4 deletion (Terker et al. 2018), suggesting a similar mechanism for the normocalciuria in these animals. Furthermore, it implies mechanisms of renal calcium handling are not entirely normal in this model even though baseline urinary calcium excretion is not different from control animals. Indeed, this was shown previously under dietary stress as WNK4^−/−^ animals, in contrast to controls, do not increase their urine calcium levels in response to a high salt, potassium deplete diet (Terker et al. 2018). In the present study, we have demonstrated that knockout animals have exaggerated calcium wasting in response to acute furosemide treatment. Bazua‐Valenti et al. recently proposed that increased calcium delivery to the DCT, as occurs following furosemide treatment, stimulates the apical calcium‐sensing receptor (CsR) to activate WNK4 and NCC, further promoting hypercalciuria (Bazúa‐Valenti et al. 2018). However, we found increased hypercalciuria following acute furosemide administration in the absence of WNK4, which is the opposite of which this mechanism would indicate. The CsR‐WNK4‐NCC mechanism is likely disabled in WNK4^−/−^ animals, given they lack WNK4 and have little functional NCC. Our finding also persisted in the presence of pharmacological NCC inhibition using hydrochlorothiazide co‐treatment. This supports the idea that our observation is not simply explained by inhibition of the CsR‐WNK4‐NCC pathway along the DCT.

The distal nephron is a heterogeneous segment comprising an initial segment, DCT1, which lacks substantial calcium reabsorption and more distal segments, DCT2 and the connecting tubule (CNT), which are the primary sites of active calcium reabsorption along the distal nephron (McCormick and Ellison, 2015). There is evidence that WNK4 is involved in the activation of TRPV5 (Jiang et al. 2007; Jiang et al. 2008; Hoover et al. 2016), the apical calcium channel mediating active reabsorption along the DCT2/CNT. Furosemide inhibition of NKCC2 increases calcium delivery to the distal nephron increasing TRPV5 mRNA and protein abundance and distal calcium reabsorption (Quamme, 1981; Lee et al. 2007) to offset calcium losses. Here, we demonstrated that WNK4^−/−^ animals have dramatically reduced TRPV5 expression along the DCT2 compared with control animals. Our data suggest this reduction in TRPV5 abundance prevents distal calcium reabsorption in WNK4^−/−^ animals leading to excess urine calcium excretion following furosemide treatment.

This finding led to the hypothesis that WNK4^−/−^ mice have a general tendency to waste calcium, if not at baseline, then under conditions that stress calcium homeostasis. However, urine calcium excretion in WNK4^−/−^ animals was not different from controls following dietary calcium deprivation. The difference between our furosemide data and our dietary data are likely explained by timing differences of the two experiments. Mechanisms underlying calcium homeostasis are multiple and involve renal and extrarenal systems. As explained above, the acute furosemide experiment is isolating a specific aspect of calcium homeostasis along the distal nephron and does not allow time for renal or extrarenal compensation. Following dietary calcium depletion, there is sufficient time for non‐WNK4 dependent systems to adjust, including those in the proximal tubule, GI tract, bone, and parathyroid gland. Our results indicate that adjustments at these sites can compensate for the lack of DCT/CNT calcium reabsorption over a 3 day‐period. Calcium deprivation of longer duration may result in total body calcium depletion at an accelerated rate in WNK4^−/−^ animals, but this remains untested. Additionally, further work is required to determine specific mechanisms of compensation along the proximal tubule and extrarenal locations. In summary, we have shown that WNK4, through TRPV5, prevents excessive calcium loss following acute furosemide administration but not during dietary calcium depletion. Our data reveal a specific role for WNK4 in renal calcium homeostasis along the distal nephron.

## Conflicts of Interest

The authors do not have any conflicts of interest to disclose.

## References

[phy214195-bib-0001] Bazúa‐Valenti, S. , L. Rojas‐Vega , M. Castañeda‐Bueno , J. Barrera‐Chimal , R. Bautista , L. G. Cervantes‐Pérez , et al. 2018 The calcium‐sensing receptor increases activity of the renal NCC through the WNK4‐SPAK pathway. J. Am. Soc. Nephrol. 29:1838–1848. 29848507 10.1681/ASN.2017111155 PMC6050918

[phy214195-bib-0002] Boros, S. , R. J. Bindels , and J. G. Hoenderop . 2009 Active Ca(2+) reabsorption in the connecting tubule. Pflugers Arch. 458:99–109. 18989697 10.1007/s00424-008-0602-6

[phy214195-bib-0003] Castaneda‐Bueno, M. , L. G. Cervantes‐Perez , N. Vazquez , N. Uribe , S. Kantesaria , L. Morla , et al. 2012 Activation of the renal Na^+^:Cl^‐^ cotransporter by angiotensin II is a WNK4‐dependent process. Proc. Natl Acad. Sci. 109:7929–7934 22550170 10.1073/pnas.1200947109 PMC3356635

[phy214195-bib-0004] Dimke, H. , J. G. Hoenderop , and R. J. Bindels . 2011 Molecular basis of epithelial Ca^2+^ and Mg^2+^ transport: insights from the TRP channel family. J. Physiol. 589:1535–1542. 21041532 10.1113/jphysiol.2010.199869 PMC3099013

[phy214195-bib-0005] Ferre, S. , J. G. Hoenderop , and R. J. Bindels . 2012 Sensing mechanisms involved in Ca^2+^ and Mg^2+^ homeostasis. Kidney Int. 82:1157–1166. 22622503 10.1038/ki.2012.179

[phy214195-bib-0006] Grill, A. , I. M. Schießl , B. Gess , K. Fremter , A. Hammer , and H. Castrop . 2016 Salt‐losing nephropathy in mice with a null mutation of the Clcnk2 gene. Acta Physiol. (Oxf) 218:198–211. 27421685 10.1111/apha.12755

[phy214195-bib-0007] Hoover, R. S. , V. Tomilin , L. Hanson , O. Pochynyuk , and B. Ko . 2016 PTH modulation of NCC activity regulates TRPV5 Ca^2+^ reabsorption. Am. J. physiol. Renal Physiol. 310:F144–151. 26608788 10.1152/ajprenal.00323.2015 PMC4719045

[phy214195-bib-0008] Jiang, Y. , W. B. Ferguson , and J. B. Peng . 2007 WNK4 enhances TRPV5‐mediated calcium transport: potential role in hypercalciuria of familial hyperkalemic hypertension caused by gene mutation of WNK4. Am. J. Physiol. Renal Physiol. 292:F545–554. 17018846 10.1152/ajprenal.00187.2006

[phy214195-bib-0009] Jiang, Y. , P. Cong , S. R. Williams , W. Zhang , T. Na , H.‐P. Ma , et al. 2008 WNK4 regulates the secretory pathway via which TRPV5 is targeted to the plasma membrane. Biochem. Biophys. Res. Commun. 375:225–229. 18703016 10.1016/j.bbrc.2008.08.010 PMC2570258

[phy214195-bib-0010] Lee, C. T. , H. C. Chen , L. W. Lai , K. C. Yong , and Y. H. Lien . 2007 Effects of furosemide on renal calcium handling. Am. J. Physiol. Renal Physiol. 293:F1231–1237. 17652376 10.1152/ajprenal.00038.2007

[phy214195-bib-0011] Mayan, H. , I. Vered , M. Mouallem , M. Tzadok‐Witkon , R. Pauzner , and Z. Farfel , et al. 2002 Pseudohypoaldosteronism type II: marked sensitivity to thiazides, hypercalciuria, normomagnesemia, and low bone mineral density. J. Clin. Endocrinol. Metab. 87:3248–3254. 12107233 10.1210/jcem.87.7.8449

[phy214195-bib-0012] McCormick, J. A. , and D. H. Ellison . 2015 Distal convoluted tubule. Compr. Physiol. 5:45–98. 25589264 10.1002/cphy.c140002 PMC5810970

[phy214195-bib-0013] Nijenhuis, T. , J. G. Hoenderop , A. W. van der Kemp , and R. J. Bindels . 2003 Localization and regulation of the epithelial Ca^2+^ channel TRPV6 in the kidney. J. Am. Soc. Nephrol. 14:2731–2740. 14569082 10.1097/01.asn.0000094081.78893.e8

[phy214195-bib-0014] Oddsson, A. , P. Sulem , H. Helgason , V. O. Edvardsson , G. Thorleifsson , G. Sveinbjörnsson , et al. 2015 Common and rare variants associated with kidney stones and biochemical traits. Nat. Commun. 6:7975. 26272126 10.1038/ncomms8975 PMC4557269

[phy214195-bib-0015] Quamme, G. A. 1981 Effect of furosemide on calcium and magnesium transport in the rat nephron. Am. J. Physiol. 241:F340–347. 7315959 10.1152/ajprenal.1981.241.4.F340

[phy214195-bib-0016] Simon, D. B. , R. S. Bindra , T. A. Mansfield , C. Nelson‐Williams , E. Mendonca , R. Stone , et al. 1997 Mutations in the chloride channel gene, CLCNKB, cause Bartter's syndrome type III. Nat. Genet. 17:171–178. 9326936 10.1038/ng1097-171

[phy214195-bib-0017] Takahashi, D. , T. Mori , N. Nomura , M. Z. H. Khan , Y. Araki , M. Zeniya , et al. 2014 WNK4 is the major WNK positively regulating NCC in the mouse kidney. Biosci. Rep. 34:195–205. 10.1042/BSR20140047 PMC4212913 24655003

[phy214195-bib-0018] Terker, A. S. , M. Castañeda‐Bueno , M. Z. Ferdaus , R. J. Cornelius , K. J. Erspamer , X.‐T. Su , et al. 2018 With no lysine kinase 4 modulates sodium potassium 2 chloride cotransporter activity in vivo. Am. J. Physiol. Renal Physiol. 315:F781–F790. 29412704 10.1152/ajprenal.00485.2017 PMC6230736

[phy214195-bib-0019] Yang, S.‐S. , Y.‐J. Hsu , M. Chiga , T. Rai , S. Sasaki , S. Uchida , et al. 2010 Mechanisms for hypercalciuria in pseudohypoaldosteronism type II‐causing WNK4 knock‐in mice. Endocrinology 151:1829–1836. 20181799 10.1210/en.2009-0951

